# Effects of ATP on Time Parameters of Contractility of Rats’ Slow and Fast Skeletal Muscles in Normal and Hypothermic Conditions

**DOI:** 10.3390/muscles2010003

**Published:** 2023-01-12

**Authors:** Adel E. Khairullin, Sergey N. Grishin, Azat I. Gabdrahmanov, Ayrat U. Ziganshin

**Affiliations:** 1 Department of Biochemistry, Kazan State Medical University, 420012 Kazan, Russia; 2 Research Laboratory of Mechanobiology, Kazan Federal University, 420008 Kazan, Russia; 3 Department of Medicinal Physics, Kazan State Medical University, 420012 Kazan, Russia; 4 Department of Pharmacology, Kazan State Medical University, 420012 Kazan, Russia

**Keywords:** fast skeletal muscle, slow skeletal muscle, hypothermia, neuromuscular synapse, ATP, P2 receptors

## Abstract

We have previously shown that hypothermia leads to an increase in the synaptic modulating effects of ATP but not of adenosine in several different animal skeletal muscles. In this paper, we studied the effect of ATP on the amplitude–time parameters of single and tetanic contractions of rats’ isolated fast (1) and slow (2) muscles at different temperatures. We found that when muscles were stimulated by the electrical field (0.1 Hz, 0.5 ms, 10 V), with a decrease in the bath temperature from 37 °C to 14 °C (3), there was an increase in the half-relaxation time of the slow muscle (*m. soleus*), but not of the fast muscle (*m. EDL*). Similar effects were observed using a carbachol-induced contraction technique, which suggests the postsynaptic (4) nature of the expansion of the contractile response of the slow muscle induced by ATP (5). To confirm the postsynaptic nature of the observed phenomenon, experiments were performed at a high calcium level (7.2 mM), in which the presynaptic effects of ATP were shown to be offset. We found that the hypercalcium condition did not significantly change the effects of ATP on the measured parameters in both muscles. To record muscle tetanic contractions, we gradually increased the frequency of electrical impulses with the increment of 2.5 Hz to achieve the fusion frequencies of 12.5 Hz for *m. soleus* and 17.5 Hz for *m. EDL* at normal temperatures. ATP (100 μM) did not change the fusion frequency for both muscles at a normal temperature but decreased this parameter for the slow muscle to 5 Hz at 14 °C without affecting that for the fast muscle. We conclude that ATP potentiates a hypothermia-induced increase in the half-relaxation time of the contraction of rats’ slow, but not fast, skeletal muscles by acting on postsynaptic P2 receptors (6).

## 1. Introduction

Ambient temperature is constantly changing and affects many body functions [1,2,3,4,5]. The daily fluctuations in the body temperature of warm-blooded animals do not exceed 3 °C (usually 0.5–1.0 °C) [6,7], and the influence of seasonal variation in environmental temperature is largely offset. Maintaining body temperature in certain values provides mammals with the ability to move and perform motor activity in a wide range of environmental temperature conditions [2].

It is known that in the absence of significant temperature fluctuations of internal organs of warm-blooded animals, peripheral parts of their bodies can experience large changes in temperature [8,9]. Thus, the peripheral skeletal muscles retain the ability to contract even when their temperature decreases significantly. According to modern concepts, the strength of skeletal muscle contraction and the rate of contraction and relaxation tend to increase with increasing temperature [10,11,12,13,14]. This was observed, for example, during physical exertion, when an increase in muscle temperature by several degrees was recorded [15,16,17]. However, the above-mentioned works did not pay much attention to the types of skeletal muscles studied. Several types of skeletal muscle fibers are distinguished, of which “slow” and “fast” are mentioned in all classifications [18,19,20]. “Slow” muscles ensure posture maintenance, while “fast” muscles perform fine movements. There is no doubt that these muscles are designed to respond differently to temperature changes, which is observed in practice [1,5,13].

The vertebrate neuromuscular synapse is known to be the best-studied cholinergic synapse. Acetylcholine (ACh) is the main neurotransmitter in the motor unit of the somatic nervous system [21,22,23]. However, embryonic myocytes also initially express receptors to purines, GABA, glutamate and glycine, as well as to ACh; in the process of development the expression of ACh receptors becomes predominant [24,25]. However, the purinergic synaptic modulation remains functionally important [26,27,28].

We have previously shown that in the neuromuscular synapse of frog phasic muscles, ATP inhibits mediator release by activating P2Y12 receptors [29,30,31]. Our further studies covered a wide range of motor unit types of different laboratory animals [32,33,34,35]. We found that the effects of ATP on the amplitude of the contractile responses of different muscles are mostly pronounced under hypothermic conditions. However, a contribution of ATP to the modulation of the temporal parameters of the contractile activity of “slow” and “fast” muscles in rodents under hypothermia has never been reported.

## 2. Results

### 2.1. Influence of Temperature on the Time Parameters of Single Contractile Responses of m. soleus and m. EDL Induced by EFS

**Figure 1:**
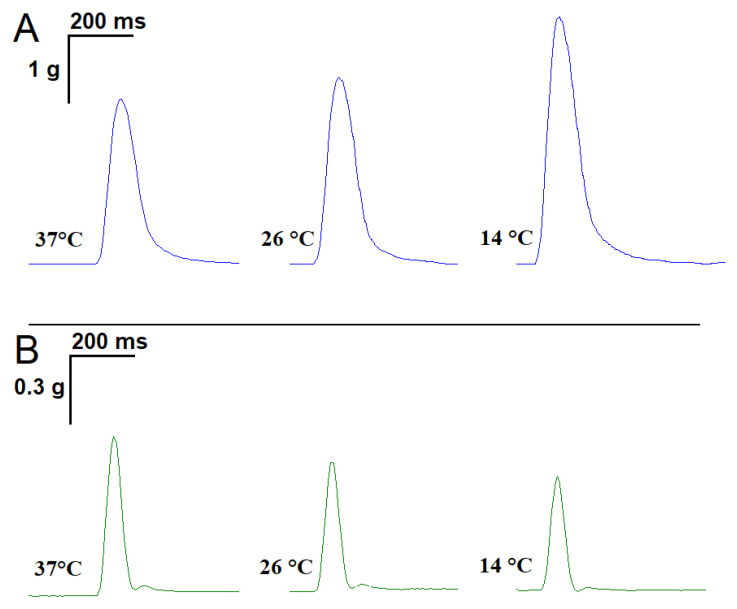
Records of single contractile responses of rat *m. soleus* (**A**) and *m. EDL* (**B**) induced by electrical field stimulation at different temperatures.

Averaged over 18 experiments, *m. soleus* contractions at 37 °C showed the following characteristics: contraction force 2.61 ± 0.07 g, contraction time 0.081 ± 0.008 s, and half-relaxation time 0.092 ± 0.007 s. With a decreasing temperature, there was an increase in both the contraction force and half-relaxation time of *m. soleus*. Thus, at 14 °C, the *m. soleus* half-relaxation time reached 0.120 ± 0.009 s (*n* = 8; *p* = 0.012; Figure 1A).

At the physiological temperature (37 °C), the contraction force of the *m. EDL* was 748.3 ± 38.9 mg, the contraction time was 0.057 ± 0.003 s, and the half-relaxation time was 0.067 ± 0.005 s. When the temperature was lowered to 14 °C, the measured parameters did not change significantly (*n* = 8; Figure 1B).

### 2.2. Effect of ATP and Suramin on Temporal Parameters of Single Contractile Responses of m. soleus and m. EDL Induced by EFS in Normal and Hypothermic Conditions

**Figure 2:**
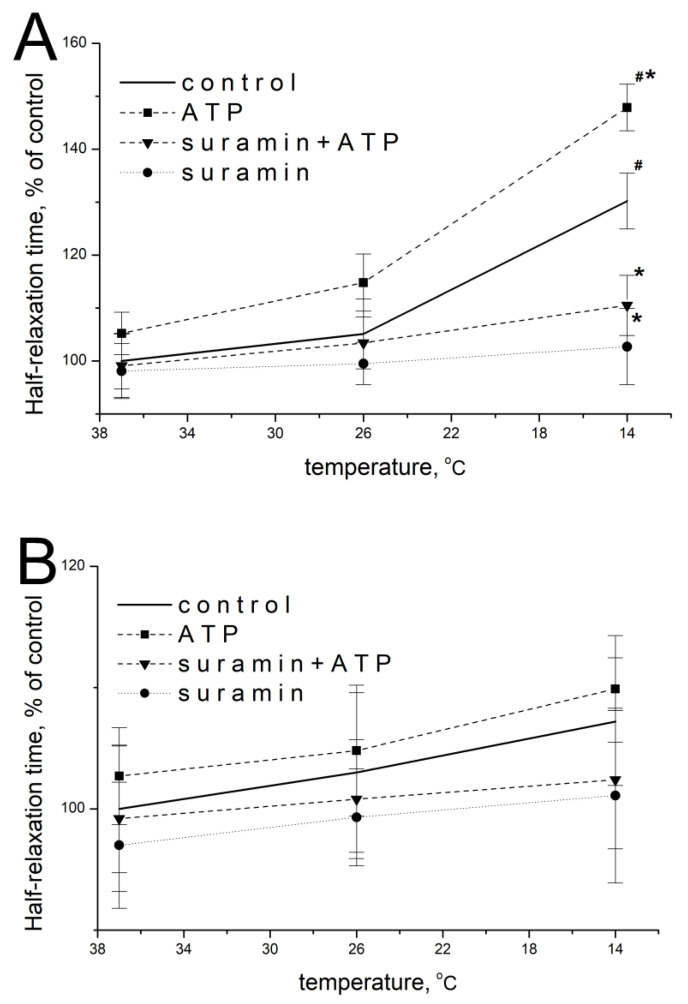
Effect of ATP (100 µM) and suramin (100 µM) on the half-relaxation time of *m. soleus* (**A**) and *m. EDL* (**B**) at different temperature regimes (*n* = 12–18). # *p* < 0.05 from the effect at 37 °C, * *p* < 0.05 from control.

ATP at a concentration of 100 μM at 37 °C did not significantly modify the parameters of EFS-evoked contractions of *m. soleus*: 105.2 ± 4.9% of initial values (*n* = 18, *p* = 0.473; Figure 2A) and 102.7 ± 6.1% (*n* = 18, *p* = 0.952; Figure 2B) for *m. EDL*.

When the temperature of the *m. soleus* incubation solution decreased, the effect of exogenous ATP was manifested by an increase in the half-relaxation time at 14 °C. Thus, at this temperature, the ATP action was 147.9 ± 5.1% (*n* = 18, *p* = 0.029, Figure 2A) of the averaged value of this parameter without ATP. The action of ATP on *m. EDL* with a decrease in temperature to 14 °C was 109.9 ± 4.7% (*n* = 18, *p* = 0.637, Figure 2B).

The modulating effect of 100 μM of ATP and temperature on both muscles at each of the temperatures studied was prevented by a non-selective P2 receptor antagonist suramin (100 μM, Figure 2A,B).

### 2.3. Effect of ATP at High Ca^2+^ Concentration

When the concentration of extracellular calcium was increased to 7.2 mM, ATP at a concentration of 100 µM at 37 °C did not significantly modify half-relaxation times of *m. soleus* and *m. EDL*, which were 105.5 ± 5.1% (*n* = 14, *p* = 0.486) and 102.9 ± 5.6% of the baseline values (*n* = 14, *p* = 0.759), respectively.

**Figure 3:**
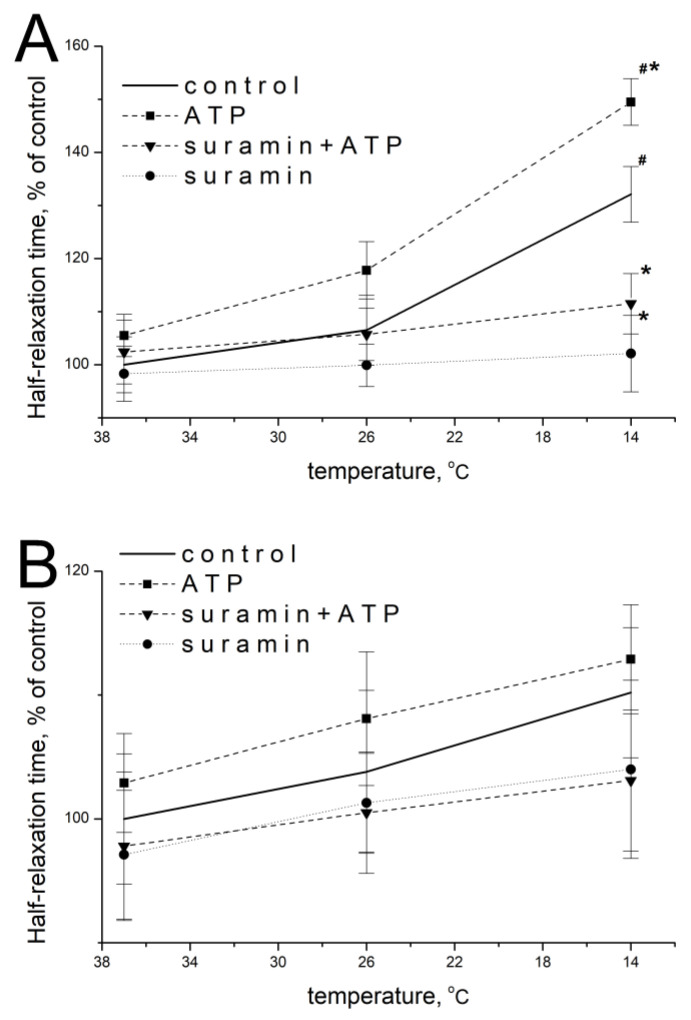
Effects of ATP (100 µM) and suramin (100 µM) on the half-relaxation time of electrical-stimulation-induced contraction of *m. soleus* (**A**) and *m. EDL* (**B**) at an elevated Ca^2+^ concentration (7.2 mM) under different temperature regimes. *n* = 8–14. # *p* < 0.05 from the effect at 37 °C, * *p* < 0.05 from control.

When the temperature decreased, the effect of exogenous ATP was manifested by an increase in *m. soleus* half-relaxation time. At 14 °C, in the presence of ATP, the half-relaxation time of this muscle was 149.5 ± 5.7% (*n* = 12, *p* = 0.022; Figure 3A), compared with the value without ATP at 37 °C. In *m. EDL*, ATP caused no significant changes: at 14 °C, ATP increased the half-relaxation time to 112.9 ± 4.6% (*n* = 12, *p* = 0.824; Figure 3B) of the value of this parameter without ATP at 37 °C. The addition of suramin at a concentration of 100 µM abolished the modulating effects of ATP and temperature in both muscles in each of the temperature regimes studied (Figure 3A,B).

### 2.4. Effect of ATP on the Tetanic Contractile Responses of m. soleus and m. EDL Evoked by Electrical Stimulation in Normal and Hypothermic Conditions

**Figure 4:**
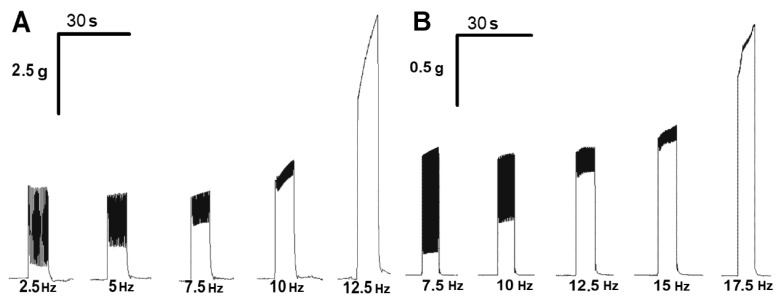
Records of contractions of *m. soleus* (**A**) and *m. EDL* (**B**) at different stimulation frequencies at 37 °C.

To record “smooth” tetanic contractions, the electrical pulse frequency was gradually increased, starting from 2.5 Hz with the increment of 2.5 Hz until single contractile responses were obtained (fusion frequency). At a normal temperature, this was observed at a frequency of 12.5 Hz for *m. soleus* and 17.5 Hz for *m. EDL* (Figure 4).

In general, ATP and suramin showed effects on the tetanic contractions of both rat skeletal muscles similar to the respective effects of these agents on the force of single-muscle contractions under the same conditions. These data are summarized in Table 1.

A decrease in the bath temperature led to a decrease in the fusion frequency of tetanic muscle contractions in *m. soleus*, but not in *m. EDL* (see Table 2). ATP at a concentration of 100 μM further decreased the fusion frequency of tetanic contractions on *m. soleus* upon hypothermia. However, on *m. EDL*, these effects were not evident.

### 2.5. Influence of Temperature on the Timing of Single Contractile Responses of m. soleus and m. EDL Induced by Carbachol

The submaximal concentration of carbachol-induced contractions of the *m. soleus* was 8 × 10^−4^ M, and for the rat *m. EDL*, it was 2 × 10^−4^ M. We found that at 20 min intervals between contractions, the muscle preparations were able to develop identical isometric contractions throughout the experiment (2–4 h).

**Figure 5:**
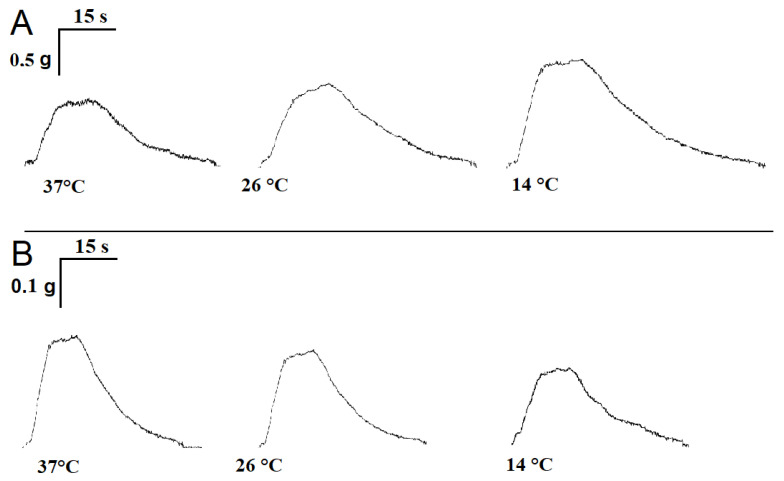
View of single contractile responses of *m. soleus* (**A**) and *m. EDL* (**B**) induced by carbachol stimulation at different temperatures.

At the physiological temperature (37 °C), the contraction parameters of *m. soleus* averaged over 18 experiments were as follows: contraction force 0.68 ± 0.08 g, contraction time 13.18 ± 0.61 s, and half-relaxation time 15.05 ± 0.74 s. For *m. EDL*, the contraction force was 187.2 ± 9.9 mg, the contraction time was 8.24 ± 0.43 s, and the half-relaxation time was 11.09 ± 0.68 s. With decreasing temperature, there was an increase in the half-relaxation time of *m. soleus*; at 14 °C, it was 20.31 ± 0.82 s (*n* = 10; *p* = 0.027; Figure 5A). The half-relaxation time of *m. EDL* did not significantly change with the decreasing temperature and was 12.08 ± 0.71 s at 14 °C (*n* = 10; *p* = 0.341; Figure 5B).

### 2.6. Effect of ATP and Suramin on Temporal Parameters of Single Contractile Responses of m. soleus and m. EDL Induced by Carbachol in Normal and Hypothermic Conditions

**Figure 6:**
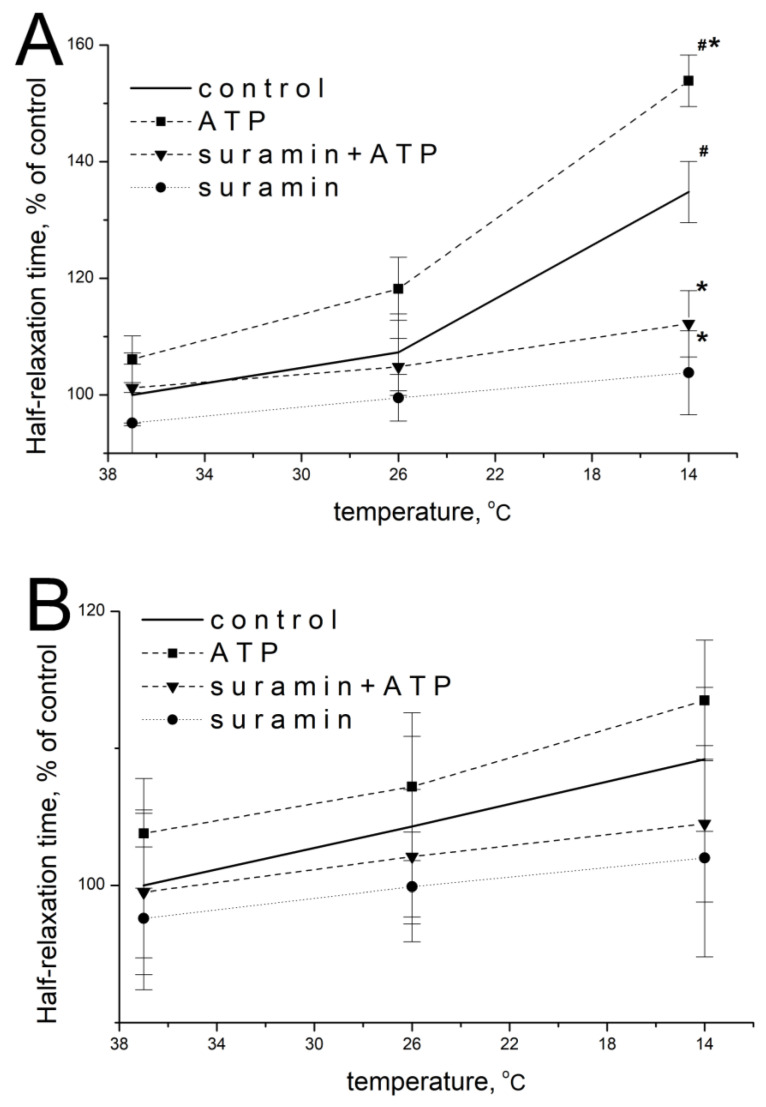
Effect of ATP on the half-relaxation time of *m. soleus* (**A**) and *m. EDL* (**B**) induced by carbachol under different temperature regimes. *n* = 9- 15. # *p* < 0.05 from the effect at 37 °C, * *p* < 0.05 from the control.

ATP at a concentration of 100 μM at 37 °C did not significantly modify the temporal parameters of carbachol-induced contractions of *m. soleus*: 106.1 ± 5.5% of baseline values (*n* = 15, *p* = 0.516; Figure 6A), and 103.8 ± 5.3% (*n* = 15, *p* = 0.713; Figure 6B) for *m. EDL*.

When the temperature of the *m. soleus* washing solution decreased, the effect of exogenous ATP at a concentration of 100 μM was manifested by an increase in the half-relaxation time at 14 °C. Thus, at this temperature, the action of 100 µM of ATP was 153.9 ± 6.4% (*n* = 9, *p* = 0.018, Figure 6A) of the averaged value of this parameter before ATP delivery. The effect of ATP on *m. EDL* with a decrease in temperature to 14 °C was 113.5 ± 5.3% (*n* = 9, *p* = 0.582, Figure 6B).

The modulating effect of ATP and temperature on both muscles at each of the temperature regimes studied was abolished by suramin, a non-selective P2-receptor antagonist (Figure 6A,B).

## 3. Discussion

It has long been known that a change in temperature has a dramatic effect on the nature of limb muscular activity. One of the earliest available scientific papers on the subject [36] begins with the following phrase: “It is well known that the contractile act is less prolonged in time at higher temperature than at lower temperature, but the effect of temperature on the mechanics of muscle contractility remains unclear”. This question remains almost just as unclear at present [37,38]. At the same time, if a muscle is stimulated directly, a change in temperature does not have such an effect. So, the cause of the discussed phenomenon is synaptic.

It was believed that changes in neuro-muscular conductivity at low temperature can be due to the dysfunction of enzyme systems [39,40] or processes of energy production and transfer [41,42]. However, it was found that the contribution of the temperature sensitivity of metabolic processes in muscle fibers cannot justify huge changes in the character of a contraction of the entire muscle organ during temperature changes [43].

Now, it has become clear that such dramatic changes in contractile responses under hypothermia are due to the involvement of ATP, a modulator of synaptic transmission.

Responses mediated by P2 receptors are temperature-dependent [44,45]. This was first established on smooth muscles [46], and later, on frog skeletal muscles [47].

In comparative experiments involving hypothermia, we further investigated the pre- and postsynaptic effects of ATP on the amplitude parameters of single contractile responses of fast and slow skeletal muscles in warm-blooded animals [31,32,33,34]. In experiments on rat muscles, it was found that ATP has negative presynaptic modulating effects in the neuromuscular junction of fast and slow muscles and postsynaptic effects in fast muscles. As for the postsynaptic effect in slow muscles, a facilitating effect of ATP on the amplitudes of contractile responses was recorded, which was most pronounced under hypothermia conditions (14 °C) [31].

In the experiments presented in this paper, a decrease in temperature led to an increase in the half-relaxation time of the rats’ slow muscle (*m. soleus*) but not the fast (*m. EDL*). Carbachol-induced contractions were used to reveal the mechanism, and similar effects were observed, suggesting the postsynaptic nature of the expansion of the contractile response of slow muscles under the influence of ATP.

Although temperature reduction significantly reduces the production of ACh in myoneural synapses, it has been shown on rat diaphragm preparations that contractions of mixed muscles are maintained and facilitated by the inhibition of the enzymatic breakdown of the mediator and the hypersensitivity of postsynaptic cholinoreceptors [48]. Similarly, in our previous experiments on slow muscle preparations, the amplitudes of contractions of rat *m. soleus* muscles increased during hypothermia, regardless of whether it was caused by the direct (carbachol) or indirect (electrical stimulation) activation of postsynaptic receptors [31]. We hypothesize that these effects are mainly due to the increased sensitivity of nicotinic receptors under hypothermia. A decrease in the activity of the mediator-degrading enzyme is probably less important here, as carbachol is less actively hydrolyzed by cholinesterase.

The nicotinic receptor has been found to contain ATP-binding centers [49,50]. ATP has also been found to increase the open state time and the frequency of opening of ACh-activated ion channels in embryonic muscles [51,52]. There is evidence that ATP may also exhibit a potentiating effect in mature muscles [53,54]. 

It has also previously been found that the inhibition of acetylcholinesterase slows down the single endplate current, indicating a postsynaptic potentiating effect. In this situation, exogenous ATP acts on current decay similarly to exogenous ACh, which can also cause the deceleration of synaptic signal decay [53].

To record “smooth” tetanic contractions, we increased the frequency of electrical impulses to 12.5 Hz for *m. soleus* and to 17.5 Hz for *m. EDL*. ATP at the reference temperatures (37, 26, and 14 °C) was found to show effects on the temporal parameters of tetanic contractions. A decrease in the rate of slow muscle fusion was observed, but not fast muscle fusion in the presence of ATP. To confirm the postsynaptic nature of the observed phenomenon in slow muscle, experiments were performed under conditions of elevated calcium (7.5 mM), which have been shown [29] to offset the presynaptic effects of ATP.

The anterior front of the smooth tetanus of both muscles recorded under in vitro conditions was of a ‘classical’ shape: it had a constant increase in strength around the ‘plateau’ phase [55].

On the other hand, in the series with smooth tetanus of both muscles used, we obtained the effects of purine agents similar to those observed in experiments with single-muscle contractions. This allowed us to fully correlate the results of the key series of experiments on electrically induced single-muscle contractions and carbacholine-induced ‘patterns’ similar in nature to tetanus [55].

We propose that the strong facilitatory postsynaptic effect exhibited by ATP, masking the weaker presynaptic effect, with decreasing temperature, allows the performance of basic locomotion even in animals subjected to deep chilling. There is recently published data on the performance of pronounced movements by the flounder muscle in animals even in the hibernation state, comparable to those observed in active individuals [54].

In the experimental results presented here, suramin prevented all changes in the amplitude–time parameters of the contraction of the rats’ soleus muscles observed during temperature reduction. Thus, neither the strength of contraction nor the time of half-relaxation of the studied muscles changed in the presence of this substance. Suramin eliminated the effect of both ATP itself and, partially, hypothermia. The trypanocidal drug suramin has a wide range of pharmacological effects [56], including the non-selective inhibition of P2 receptors [57] and the suppression of several families of ecto-nucleotidases [58,59]. In our experiments, it not only counteracted the effects of ATP on the contractile parameters of rat muscles but also prevented hypothermia-dependent changes in contractility—regardless of the type of stimulation. This may imply that, as in other organs [60], there is a natural mechanism in warm-blooded skeletal muscles by which P2 receptors mediate temperature changes that become more prominent at low temperatures and which are almost completely inhibited by suramin.

So, why is the post-synaptic effect of ATP on the expansion of the contractile response evident in the synapse of the slow muscle? It is known that the synaptic area is twice as large in the *m. soleus* than in the *m. EDL* [61].

The possibility of reciprocal shifts of ion channels floating freely in the lipid phase of the chemoexcitable cell membrane due to the ampere interaction of the currents flowing through them is also known [62]. Although these forces are extremely weak, according to simulations, in the absence of thermal motion and long-term synaptic activity, they could lead to changes in the spatial interposition of ion channels. Ampere forces under hypothermia can initiate the clustering of acetylcholine-activated receptor–channel complexes in regions of the postsynaptic membrane located opposite the active zones of the presynaptic membrane, or in other words, opposite the sites of mediator quantum release. Additionally, it is in the slow muscle that this occurs preferentially in the larger area of the synapse.

Based on the above data, we suggest that the temperature-sensitive tonic effects of endogenous ATP on the force and half-relaxation time of contraction underlie the phenomenology of changes in muscle responses during temperature reduction.

## 4. Materials and Methods

### 4.1. Animals and Surgery

The studies were carried out on neuromuscular preparations of white male laboratory rats weighing 140–180 g, which were kept in groups of 3–5 individuals with water and food ad libitum. A total of 108 rats were used in these studies. The animals were anaesthetized via the intraperitoneal administration of sodium ethaminal at a dose of 40 mg/kg, exsanguinated, and *m. soleus* and *m. EDL* were isolated on both hind limbs. The isolated muscles were fixed vertically with one end fixed and another attached to a mechanical activity sensor and immersed in 10 mL baths filled with Krebs solution of the following composition (in mM): NaCl 118.0, KCl 4.75, CaCl_2_ 2.5, NaHCO_3_ 24.8, KH_2_PO_4_ 1.18, MgSO_4_·7H_2_O 1.18, glucose 11, pH = 7.4, t = 37 ± 0.5 °C. The thermostat maintained the set point temperature. Muscles were strained with an initial load of 1 g, then left at rest for 30 min to get used to the environmental condition.

### 4.2. Single Contractions Induced by Electrical Field Stimulation

Electrical field stimulation (EFS) was performed by stimulating the nerve stump, which was placed in a suction-electrode of the original design. A Digitimer MultiStim D330 stimulator (UK) was used for stimulation. Muscle contractions were induced by stimulation with rectangular pulses of 0.1 Hz frequencies, 0.5 ms lengths, and 10 V amplitudes for 2 min. The contraction parameters were recorded with a Linton FSG-01 isometric mechanical activity sensor (UK), and the analog signal was digitized and processed using a Biopack MP100WSW data acquisition system (USA). The average value of parameters of all contractions obtained during 2 min (12 responses) was processed as one result. Parameters of contractile responses were calculated in % relative to initial results obtained at the beginning of the experiment.

After 30 min of tissue fixation, control stimulation of the muscle was performed twice at 5 min intervals, and after ensuring the stability of the contractile responses, experimental procedures were started.

After the initial control responses of the muscle were recorded, ATP (100 µM) was added to a bath, and the contractile responses of the muscle induced by EFS were evaluated after 10 min. After washout, the tissue was incubated with suramin (100 µM) for 20 min, followed by the addition of ATP (100 µM) for 10 min, and the contractile responses were recorded again.

**Figure 7:**
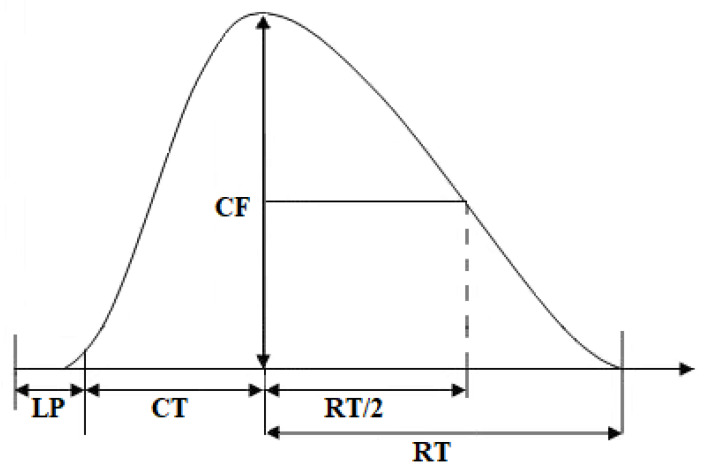
Schematic diagram of recording contractile parameters. CF—contraction force, LP—latency period, CT—contraction time, RT—relaxation time, RT/2—half-relaxation time.

The contractile properties of isolated neuromuscular preparations were studied according to three parameters, which allowed a sufficiently complete reflection of the functional activity of the studied skeletal muscles: contraction time, half-relaxation time (both in seconds), and contraction force (in g), which were determined by analyzing the contraction curve of the studied muscle (Figure 7).

### 4.3. Contractions Induced by High-Frequency Electrical Field Stimulation

To record “smooth” tetanic contractions, the frequency of EFS impulses was gradually increased with the increment of 2.5 Hz to achieve the fusion frequency. Muscle stimulation was performed in short bursts of pulses for 7 s. At these frequencies, single-muscle contractions of the corresponding muscles were not distinguished, i.e., “smooth” tetanus was observed [54]. The mean value of the contraction force was defined as the average amplitude point of the plateau phase in 2 min, and the force of the contraction was determined in milligrams. All data obtained were calculated in % relative to the initial results obtained at the beginning of the experiment at 37 °C.

In the control experiments, the tissue was incubated with suramin (100 µM) for 20 min, and the muscle responses to EFS were recorded. In each series of the experiments, the effects of the agonists and antagonists were also evaluated in the presence of d-tubocurarine, a nicotinic cholinoblocker, at a concentration of 10 µM.

### 4.4. Carbachol-Induced Contractions

The contractile responses were induced by adding submaximal concentrations of carbachol to the bath. Experiments were carried out to determine the concentration dependence of carbachol on the test tissue. The submaximal concentration of carbachol was calculated to give 70% of the maximum possible contraction force (usually 3–10 µM). Based on the experiments performed, contractile characteristics (contraction force, contraction time, and half-relaxation time) were obtained under different temperature conditions in the presence of ATP (100 µM) and suramin (100 µM). The mean value of contraction force was treated as a single measurement.

### 4.5. Assessment of the Temperature Dependency of Rat Skeletal Muscle Contraction

The effect of temperature on the contraction parameters of *m. soleus* and *m. EDL* was evaluated in experiments with both EFS and carbachol application initially at 37 °C. The temperature was then decreased to 26 °C and 14 °C. At each temperature condition, ATP (100 M) was applied, and the contractile responses of the muscle were evaluated after 10 min of application. The tissue was then incubated with suramin (100 µM) for 20 min, followed by the addition of ATP (100 µM). The temperature of the solution was controlled with a TE-8A water pump (Techne, UK), and a rapid decrease in the temperature of the liquid in the water pump was performed by adding ice.

### 4.6. Experiments in Hypercalcium Environment

In separate series of the experiments with EFS, the muscle responses to ATP and suramin at different temperature conditions were recorded first in normal Krebs solution and then in a solution with an elevated calcium ion concentration, 7.2 mM.

### 4.7. Data Analysis

Mechano-myographic experiments on *m. soleus* and *m. EDL* rats were analyzed using ANOVA. A P level of less than 0.05 was taken as significant. Experimental data are presented as mean ± standard error of the mean (*n* is the number of neuromuscular preparations for the mechano-miographic experiments).

## Figures and Tables

**Table 1:** Effect of ATP (100 µM) and suramin (100 µM) on the contraction force of the tetanic contraction (in %) of *m. soleus* (A) and *m. EDL* (B) under different temperature regimes (*n* = 8). * *p* < 0.05 from the effect at 37 °C, # *p* < 0.05 from control).

Muscles	Substances	Temperature		
		37 °C	26 °C	14 °C
*m. soleus*	Control	100	112 ± 7.1	153 ± 5.9 *
	ATP	69 ± 8.0 #	82 ± 7.7 #	157 ± 9.5 *
	suramin + ATP	103 ± 5.7	95 ± 6.4	107 ± 6.8 #
*m. EDL*	Control	100	79 ± 5.8	64 ± 7.2 *
	ATP	86 ± 5.5 #	58 ± 7.9 #	43 ± 6.2 *#
	suramin + ATP	98 ± 7.1	97 ± 8.5	94 ± 9.1 #

**Table 2:** Effect of temperature on the fusion frequency of tetanic muscle contractions (in Hz) in control and with application of ATP and suramin at a concentration of 100 µM. *n* = 12.

Muscles	Substances	37 °C	26 °C	14 °C
*m. soleus*	Control	12.5	10	7.5
	ATP	12.5	7.5	5
	suramin	12.5	10	10
	suramin + ATP	12.5	10	7.5
*m. EDL*	Control	17.5	17.5	15
	ATP	17.5	17.5	15
	suramin	17.5	17.5	15
	suramin + ATP	17.5	17.5	15

## Data Availability

Not applicable.
